# HIPK2 Is Required for Midbody Remnant Removal Through Autophagy-Mediated Degradation

**DOI:** 10.3389/fcell.2020.572094

**Published:** 2020-09-15

**Authors:** Francesca Sardina, Laura Monteonofrio, Manuela Ferrara, Fiorenza Magi, Silvia Soddu, Cinzia Rinaldo

**Affiliations:** ^1^Institute of Molecular Biology and Pathology (IBPM), National Research Council (CNR), c/o Sapienza University of Rome, Rome, Italy; ^2^Unit of Cellular Networks and Molecular Therapeutic Targets, IRCCS Regina Elena National Cancer Institute, Rome, Italy

**Keywords:** HIPK2, midbody remnants, abscission, autophagy, nbr1

## Abstract

At the end of abscission, the residual midbody forms the so-called midbody remnant (MBR), a platform affecting cell fate with emerging key role in differentiation, development, and tumorigenicity. Depending on cell type and pathophysiological context, MBRs undergo different outcomes: they can be retained, released, internalized by nearby cells, or removed through autophagy-mediated degradation. Although mechanisms underlying MBR formation, positioning, and processing have been recently identified, their regulation is still largely unknown. Here, we report that the multifunctional kinase HIPK2 regulates MBR processing contributing to MBR removal. In the process of studying the role of HIPK2 in abscission, we observed that, in addition to cytokinesis failure, HIPK2 depletion leads to significant accumulation of MBRs. In particular, we detected comparable accumulation of MBRs after HIPK2 depletion or treatment with the autophagic inhibitor chloroquine. In contrast, single depletion of the two independent HIPK2 abscission targets, extrachromosomal histone H2B and severing enzyme Spastin, only marginally increased MBR retention, suggesting that MBR accumulation is not just linked to cytokinesis failure. We found that HIPK2 depletion leads to (i) increased levels of CEP55, a key effector of both midbody formation and MBR degradation; (ii) decreased levels of the selective autophagy receptors NBR1 and p62/SQSTM1; and (iii) impaired autophagic flux. These data suggest that HIPK2 contributes to MBR processing by regulating its autophagy-mediated degradation.

## Introduction

During the final stage of cell division, daughter cells are connected by an intercellular bridge that contains bundled microtubules that overlap in an antiparallel manner at the midbody. The midbody is an electron-dense structure that works as a platform for the spatiotemporal distribution of specific proteins and lipids that contribute to abscission, the final cut when the intercellular bridge is severed (Hu et al., 2012). In addition to its canonical role to orchestrate abscission, accumulating evidence has indicated postabscission signaling roles of the midbody remnant (MBR), also named midbody derivative, midbody ring derivative, postcytokinesis, or postabscission or postmitotic midbody (Chen et al., 2013; Peterman and Prekeris, 2019). Following abscission, some of the midbody-localized factors, such as the ESCRT-III recruiters CEP55 and ALIX, persist at the MBR together with several signaling proteins (Willard and Crouch, 2001; Kaplan et al., 2004; Naito et al., 2006; Cho and Kehrl, 2007; Kasahara et al., 2007; Fumoto et al., 2012; Chen et al., 2013). Depending on the cell type and tissue context, MBRs may be released in the extracellular space, inherited by one of the dividing cells, or internalized by interphase cells (Chen et al., 2013; Crowell et al., 2014; Peterman and Prekeris, 2019). These different MBR outcomes have functional consequences in cell-fate determination with key roles in differentiation and development, while their defects can be implicated in different pathological conditions. For example, in neuronal and epithelia morphogenesis, a crucial role is played by MBR positioning that determines cell polarity in neurite outgrowth and in apical lumen formation (Wilcock et al., 2007; Pollarolo et al., 2011; Li et al., 2014; Luján et al., 2016), and such polarity cues have been shown to be required also for ciliogenesis (Bernabé-Rubio et al., 2016). A different example is represented by asymmetrically dividing stem cells and cancer cells in which MBR function is linked to its processing. In these cells, MBRs are long-lived structures that can persist in the cytoplasm for hours mainly due to evasion from autophagic degradation, which is mediated by the binding of the autophagic receptor NBR1 to its ligand CEP55, a key component of both midbodies and MBRs (Kuo et al., 2011; Crowell et al., 2013). In addition to receptor recognition through NBR1/CEP55 complex, MBRs have been shown to be degraded through selective autophagy, a process called autophagy, in which the autophagic factors p62/SQTM1, ALFY, TRAF6 are involved, with the contribution of the E3 ubiquitin-ligase TRIM17 and the autophagy adaptor FYCO-1 (Isakson et al., 2013; Mandell et al., 2016; Dionne et al., 2017). At the functional level, MBR accumulation has been observed in cancer cells and proposed to regulate cell stemness, proliferation, and tumorigenicity. In particular, MBR-enriched subpopulations of cancer cells show higher tumorigenic potential characterized by increase in proliferation, anchorage independent growth, and invadopodia formation (Kuo et al., 2011; Dionne et al., 2017; Peterman et al., 2019). Despite the functional consequences of retention and signaling of MBRs, how their degradation is regulated is poorly understood.

HIPK2 is an evolutionary conserved multifunctional serine/threonine kinase (Calzado et al., 2007; Rinaldo et al., 2007; D’Orazi et al., 2012; Blaquiere and Verheyen, 2017), whose interactors and targets belong to several signal transduction pathways affecting cell fate. HIPK2 is involved in crucial processes, such as development, differentiation, and response to DNA damage and oxidative stress (Calzado et al., 2007; Rinaldo et al., 2007; D’Orazi et al., 2012; Blaquiere and Verheyen, 2017). We previously demonstrated that HIPK2 localizes at the midbody in an Aurora-B–dependent manner and is among the kinases that regulate abscission (Rinaldo et al., 2012; Monteonofrio et al., 2018). In particular, HIPK2-depleted or -null cells undergo abscission delay and failure, accumulating elongated midbodies and binucleated cells, whose uncontrolled proliferation leads to chromosomal instability and increased tumorigenicity (Rinaldo et al., 2012; Valente et al., 2015). Mechanistically, HIPK2 regulates abscission with two apparently parallel mechanisms of action. HIPK2-mediated phosphorylation of the extrachromosomal histone H2B (ecH2B) at Ser14 is not required for its midbody localization but contributes to the formation of the abscission site and is essential for successful cytokinesis (Rinaldo et al., 2012; Monteonofrio et al., 2019). HIPK2-mediated phosphorylation of the microtubule severing enzyme Spastin at Ser268 is required for its midbody localization to cut microtubules and the subsequent physical separation of the two daughter cells (Pisciottani et al., 2019; Gatti et al., 2020).

During our studies on HIPK2 abscission functions, we noticed that HIPK2-depleted cells show a significant accumulation of MBRs. By comparing cells treated with the autophagic inhibitor chloroquine (CQ) or depleted with siRNAs specific for HIPK2 abscission targets, we showed that HIPK2 regulates MBR degradation by controlling CEP55 levels and the autophagic pathway.

## Materials and Methods

### Cells, Culture Conditions, and Treatments

HeLa (a gift of N. Corbi), U2OS (a gift of F. Moretti), HeLa^Ctr–Cas9^, and HeLa^HIPK2–Cas9^ (Ritter and Schmitz, 2019) (a gift M.L. Schmitz) and hTERT RPE-1 (a gift of G. Guarguaglini) were cultured at 5% CO_2_ and 37°C in Dulbecco modified eagle medium (DMEM) GlutaMAX supplemented with 10% heat-inactivated fetal bovine serum (FBS) (Life Technologies); mouse motoneuron-like NSC34 cells (a gift of M. Cozzolino) were cultured in DMEM-F12 1:1, supplemented with 10% FBS. Cells were routinely analyzed for mycoplasma contamination. Treatment with CQ (Sigma–Aldrich) or its solvent phosphate-buffered saline (PBS) (Life Technologies) were performed at 10 mM for 72 h.

Brain tissues were explanted from *Hipk2* KO adult mice carrying first conditional-ready allele and their wild-type (WT) counterpart.

### RNA Interference and Expression Vector Transfection

In human cells, HIPK2 RNA interference (RNAi) and Spastin RNAi were obtained by using stealth siRNAs as in Pisciottani et al., 2019. Specific prevalidated stealth siRNAs by Life Technologies were used to obtain HIPK2 RNAi in mouse cells and CEP55 RNAi in human cells. In details, cells were transfected with a mix of at least two stealth siRNAs at a final concentration of 40 nM using Lipofectamine RNAi MAX (Life Technologies). Depletion of ecH2B was obtained as in Monteonofrio et al., 2019. Briefly, cells were transfected with a mix of nine siRNAs each at 10 nM in a double pulse, the second one 24 h after the first one. As control, stealth siRNA Negative Medium GC Duplex (Life Technologies) was used with the respective transfection strategies, i.e., single- or double-pulse transfection and siRNA concentration.

Expression vectors were transfected by using Lipofectamine LTX and Plus reagent (Life Technologies). For rescue experiments, human HeLa siHIPK2 cells were transfected with low doses of vectors expressing GFP-tagged murine HIPK2-WT (D’Orazi et al., 2002) or its empty vector (peGFP-c2; Clontech) as in Pisciottani et al., 2019. Murine HIPK2 protein shows >97% identity with human HIPK2, fully recapitulates its functions, but is resistant to RNAi when human-specific siRNAs were used.

### Immunofluorescence Analysis

Cells were seeded onto poly-L-lysine–coated coverslips, fixed in 2% formaldehyde or in ice-cold methanol, permeabilized in 0.25% Triton X-100 in PBS for 10 min, and then blocked in 5% bovine serum albumin in PBS for 1 h before the primary antibody (Ab) was applied. Employed Abs were as follows: anti-ALIX (1:100; #sc-53538; Santa Cruz Biotechnology), anti-CEP55 (1:700; # 00055165-A01; Bionova); anti–β-tubulin-Cy3 (Sigma–Aldrich), secondary 488- or 594-conjugated Abs (1:300; Alexa-Flour, Life Technologies). DAPI (Sigma–Aldrich) was used to stain DNA. Cells were examined under an upright Olympus BX53 microscope equipped with a Lumen 200 Fluorescence Illumination System (Prior Scientific) with a 200-W metal arc lamp, and photographs were taken (× 100 or × 60 objectives) using a cooled camera device (ProgRes MF). Images for each sample were taken selecting the appropriate Olympus filters (DAPI: U-MNU2; FITC: U-MNB2; Texas red: U-MWIY2) at 100% of excitation light intensity with different exposure time for tubulin, CEP55, ALIX, and DAPI, respectively, of 500, 640, 1,264, and 16 ms. For MBR quantification, because CEP55 and ALIX also label centrosome, bonafide MBRs were considered only those clearly showing costaining of CEP55 or ALIX with β-tubulin that is strongly enriched in this subcompartment.

### Western Blot

Total cell extracts (TCEs) were prepared in denaturing buffer (50 mM Tris–HCl pH 8.0, 600 mM NaCl, 0.5% sodium deoxycholate, 1% NP40 0.1% sodium dodecyl sulfate, and 1 mM EDTA) supplemented with protease and phosphatase inhibitors (Roche). Proteins were resolved using precast Bolt Novex Bis–Tris Gels 4–12% (Life Technologies), transferred to nitrocellulose membranes (Bio-Rad), and immunoreactivity was determined using ECL (Amersham). Acquisition and densitometric analysis of images were obtained using Image Lab software (Bio-Rad). Employed Abs were as follows: anti-HIPK2 (rat monoclonal Ab C5C6 kindly provided by M. L. Schmitz); anti-Spastin (1:100; #sc-53443), anti-GAPDH (1:1,000; #sc-32233), anti–α-tubulin (1:1,000; #sc-5286), anti-actin (1:1,000; #sc-47778), anti-ALIX (1:200; #sc-53538), anti-p62 (1:1,000; #28359), anti-lamina A/C (1:1,000; #sc-20680) by Santa Cruz Technology; anti-CEP55 (1:1,000; #00055165-A01), and anti-NBR1 (1:500; #H00004077-B01P) by Abnova; anti-TSG101 (1:500: #ab30871) and anti-H2B (1:1,000; #ab52484) by Abcam; anti-LC3 (1:1,000; #L8918 by Sigma–Aldrich) recognizing both the cytosolic LC3-I and lipidated LC3-II forms of LC3. Anti–horseradish peroxidase–conjugated goat anti–mouse #7076, anti–rabbit #7074, and anti–rat #7077 (Cell Signaling Technology).

### Autophagy Flux Analysis

Autophagic flux was determined by quantifying the autophagosome marker LC3-II in the presence of CQ to inhibit lysosomal degradation or its solvent, PBS, as described in Kuo et al., 2011. Briefly, siCtr and siHIPK2 cells were treated with CQ or PBS for 24 h, and their TCEs analyzed by Western blot (WB) with anti-LC3 Ab; GAPDH was used as loading control. Data acquisition and densitometric analyses for LC3-II and GAPDH were performed by Image Lab (Bio-Rad). Densitometric values were employed to calculate the LC3-II relative level as LC3-II/GAPDH ratio; the autophagic flux for each condition is the result of the following equation: autophagic flux = 100 - [(PBS LC3-II relative level/CQ LC3-II relative level) × 100)].

### Statistical Analysis

Each experiment has been repeated three or five times, and data analyses were performed using the GraphPad Prism software. Statistical significance was assessed by unpaired *t* test; significance was set at *p* < 0.05.

## Results

### HIPK2 Depletion Leads to MBR Accumulation

**FIGURE 1:**
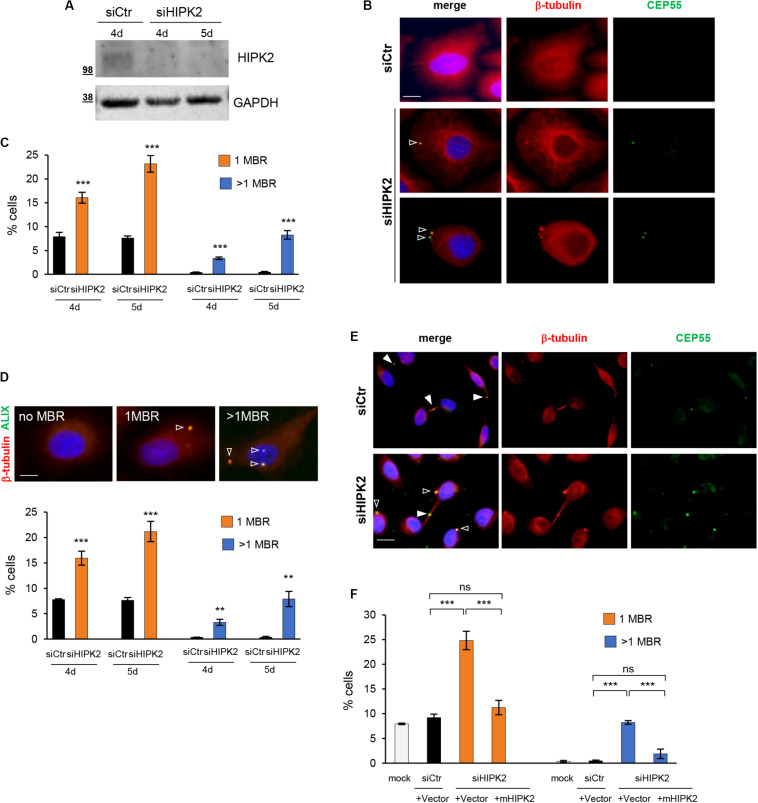
MBR in HIPK2-depleted cells. **(A–D)** HeLa cells were transfected with HIPK2 -specific siRNA or negative universal control and analyzed 4 and 5 days posttransfection by WB with indicated Abs to verify RNAi and by IF for MBR quantification by using CEP55 as MBR marker in combination with β-tubulin costaining. DAPI (blue) was used to stain DNA. Representative WB and images are shown in **(A,B)**, respectively. Scale bar, 5 μM. Here, and in the following images, the empty arrowheads indicate MBR. The percentage of cells with 1 or >1 MBR were reported as mean ± standard deviation (SD) in **(C)** from three independent experiments, in which at least total 3,000 cells per condition were analyzed. ***p < 0.001 and **p < 0.01, unpaired *t* test. **(D)** HeLa cells were transfected as in **(A)**, analyzed by IF by using ALIX as MBR marker in combination with β-tubulin costaining and DAPI to stain DNA. Data reported as in **(C)**, are from three independent experiments, in which at least total 3,000 cells per condition were analyzed. ***p < 0.001, **p < 0.01, unpaired *t* test. Representative images are shown. Scale bar, 5 μM. **(E)** Representative images of siCtr and siHIPK2 HeLa cells 5 days posttransfection. CEP55 marks MBR as well as the midbody. Here, and in the following images, the full arrowheads indicate CEP55 at midbody. Scale bar, 10 μM. **(F)** HeLa cells 36 h upon transfection with low doses of vectors expressing GFP-tagged murine HIPK2-WT or GFP empty vector and analyzed by IF as in **(C)**. Data are reported as mean ± SD from three independent experiments, in which a total of 2,000 cells per condition were analyzed. Note that the expression of murine HIPK2 protein is not silenced by RNAi because human-specific siRNAs were used in siHIPK2 cells. ***p < 0.001, ns = not significant, unpaired *t* test.

To characterize HIPK2 functions in abscission, we previously examined the microtubule stability and the localization of several cytokinesis factors at the intercellular bridge by immunofluorescence (IF) in cells treated with control siRNA (siCtr) or a mix of HIPK2-specific siRNAs (siHIPK2). To evaluate the effect of HIPK2 depletion over more than one cell cycle, cells were transfected with stealth siRNAs that have increased stability in cell culture than standard siRNAs. We showed that HIPK2 depletion delays abscission and induces accumulation of aberrant elongated midbodies and cytokinesis failure (Rinaldo et al., 2012; Pisciottani et al., 2019). During these studies, we also observed the appearance of a large fraction of cells with MBRs in the siHIPK2 cells compared to their relative siCtr (Figures 1A,B, and Supplementary Figure S1). Quantification of MBRs in fixed HeLa (Figure 1) and U2OS (Supplementary Figure S1) cells was performed by using the MBR marker CEP55 in combination with β-tubulin enrichment and revealed a significant increase in the percentage of cells carrying one or more than one MBR at days 4 and 5 posttransfection in the siHIPK2 cells (Figure 1C). Binucleated cells and cells with abnormally large nuclei were excluded from our analysis to avoid cells that have undergone cytokinesis failure. Similar findings were observed by using an Ab against ALIX, a protein highly concentrated in MBR (Figure 1D). Of relevance, we also noticed some MBRs still persisting in siHIPK2 cells at telophase stage (5.3% ± 1.1% in siHIPK2 cells vs. <0.5% in siCtr by analyzing 300 telophases for each condition in three independent experiments) (Figure 1E), suggesting that cells underwent new mitosis without degrading the MBR of the previous cell divisions.

Next, we restored HIPK2 expression in the siHIPK2 cells by transfecting an siRNA-resistant HIPK2 cDNA and showed inhibition of MBR accumulation (Figure 1F), excluding RNAi off-target effects.

Overall, these results show that MBRs accumulate and may even persist through successive divisions in HIPK2-depleted cells, suggesting that HIPK2 has a role in controlling MBR fate.

### MBR Accumulation Is Only Marginally Detected Upon Depletion of HIPK2 Cytokinesis Targets

**FIGURE 2:**
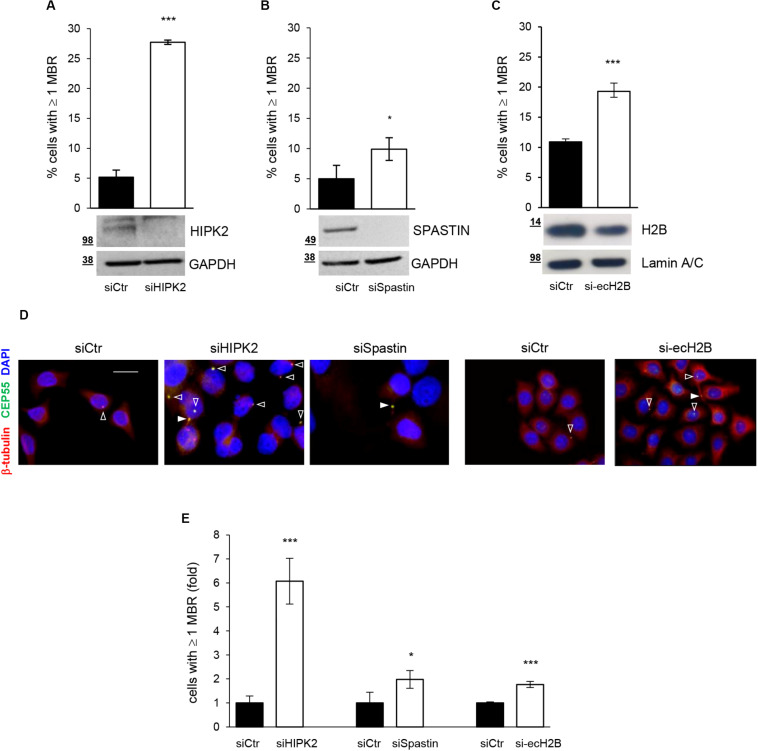
MBR upon depletion of HIPK2 cytokinesis targets. **(A–D)** HeLa cells were transfected with siRNAs specific for HIPK2, Spastin, and H2B or negative control and analyzed by WB with indicated Abs to verify RNAi and by IF to quantify MBR. MBR quantitation, performed as in Figure 1C, is reported as mean ± SD from three independent experiments in which at least 1,500 cells per condition were analyzed. Representative WBs are shown below each graph, and representative images are reported in **(D)**. Scale bar, 10 μM. **(E)** MBR quantitation is reported also as fold change relative to siCtr, which was set to 1 for each condition. ***p < 0.001 and *p < 0.05, unpaired *t* test.

HIPK2 has been shown to contribute to cytokinesis through two distinct targets, ecH2B, which contributes to the formation of the abscission site (Monteonofrio et al., 2019), and Spastin, which severs microtubules for the final cut (Pisciottani et al., 2019). Thus, we quantify MBRs also after depletion of Spastin and ecH2B obtained by transfecting HeLa cells with Spastin-specific siRNAs (siSpastin) and H2B-specific siRNAs (si-ecH2B), as reported (Monteonofrio et al., 2019; Pisciottani et al., 2019). Alongside, for each condition, we also evaluated the induction of cytokinesis defects by analyzing the percentage of aberrant midbodies and binucleated cells, as we previously reported (Rinaldo et al., 2012) (Supplementary Table S1). As shown in Figures 2A–D, compared to their relative siCtr cells, a mild, although significant, accumulation of MBRs was observed in both siSpastin and si-ecH2B cells. However, when compared to siHIPK2 cells, MBR accumulation induced by Spastin and ecH2B depletion was considerably lower (Figure 2E). Similar data were observed in human non-transformed RPE-1 cells (Supplementary Figure S2 and data not shown). These data suggest that HIPK2 depletion might either induce MBR accumulation in a manner that is largely independent of cytokinesis defects or that both Spastin and ecH2B need to be simultaneously knocked down. Because the depletion strategies for Spastin and ecH2B are not compatible (see Materials and Methods), we focused our attention on the first hypothesis.

### HIPK2 Regulates MBR Removal Through Autophagy-Mediated Degradation

**FIGURE 3:**
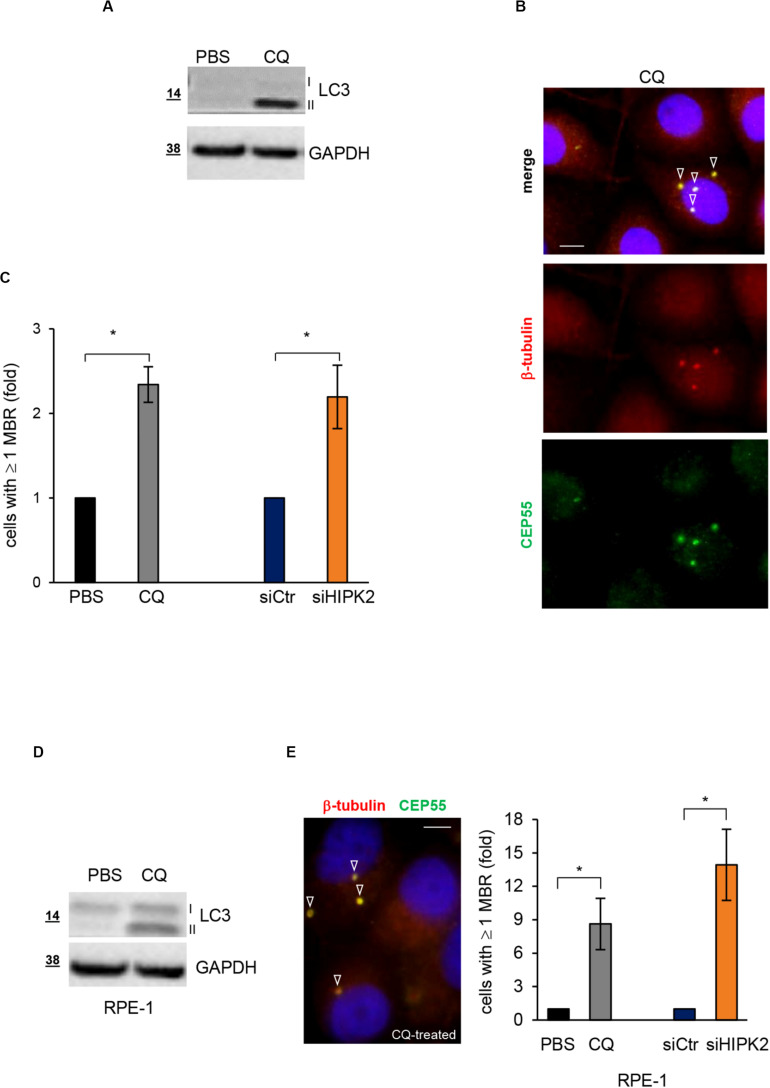
HIPK2-depleted cells show MBR comparable to those of cells treated with the autophagy inhibitor CQ. **(A–C)** HeLa cells were treated with PBS or CQ 10 mM for 3 days and analyzed to verify autophagy inhibition by WB **(A)** and MBR quantitation by IF **(B)**. WB analysis was performed with anti-LC3 Ab that recognizes both cytosolic LC3-I and lipidated LC3-II forms of LC3. IF was performed as in Figure 1B. Representative WB and images are shown in **(A,B)**, respectively. Scale bar, 5 μM. In **(C)**, the percentage of cells with ≥1 MBR relative to PBS-treated cells are reported and compared to that of siHIPK2 cells relative to siCtr measured 4 days posttransfection, performed as in Figures 1A–C. **(D,E)** RPE-1 cells were treated, analyzed, and compared to siHIPK2 as performed for HeLa cells in **(A–C)**. Representative image of CQ-treated cells is shown. Scale bar, 5 μM. *p < 0.05, unpaired *t* test.

Autophagy is the main specific degradation mechanism for MBR removal (Pohl and Jentsch, 2009; Kuo et al., 2011; Isakson et al., 2013). Recently, exogenous expression of HIPK2 in primary mouse hepatocytes has been shown to stimulate autophagy (Jiang et al., 2018). Thus, we asked whether HIPK2 might have a role in mediating MBR autophagic degradation. As a first insight, we compared siHIPK2 cells with those treated with the autophagy inhibitor CQ, which inhibits lysosomal activity allowing MBR accumulation (Pohl and Jentsch, 2009). In particular, we evaluated the effect of CQ on MBR accumulation in HeLa cells. Autophagy inhibition by CQ was confirmed by WB detection of the lipidated form of the microtubule-associated protein 1A/1B light chain 3 (LC3-II), whose accumulation is induced when the lysosomal activity is blocked, and the turnover of the LC3-II pool present in the autophagomes is prevented (Klionsky et al., 2008; Figure 3A). In the same culture conditions, the percentage of cells with ≥1 MBR was measured by IF, as above (Figure 3B). Consistent with the possibility that defects in autophagic pathways underlie the MBR accumulation in siHIPK2 cells, comparable fractions of cells with MBR accumulation were observed in CQ-impaired autophagy and HIPK2-depleted cells (Figure 3C). Similar results were observed in RPE-1 cells (Figures 3D,E).

**FIGURE 4:**
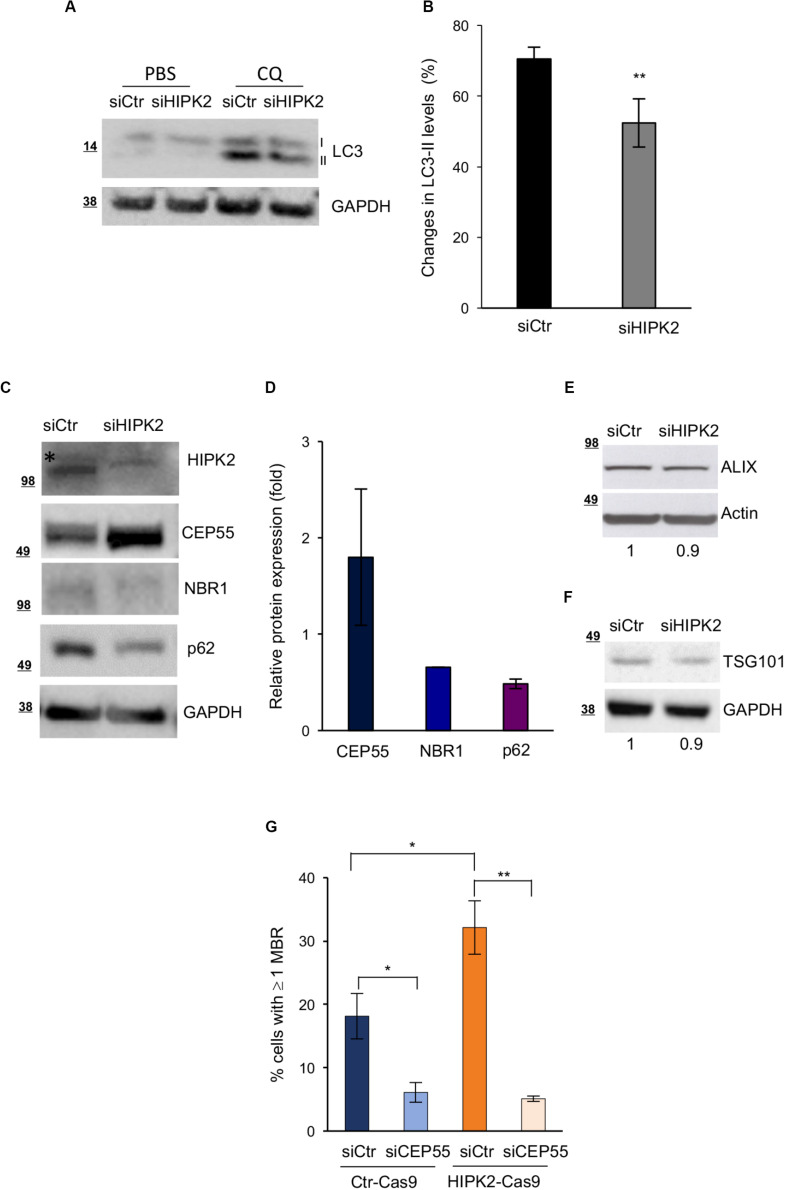
CEP55 overexpression and reduced autophagic flux after HIPK2-depletion. **(A,B)** HeLa cells were transfected as in Figure 1A and treated with PBS or CQ 48 h posttransfection. Autophagic flux was measured by WB 24 h posttreatment, scoring the changes in the levels of LC3-II, in the presence or absence of CQ. Representative WB is shown in A. Data quantification of four experiments is reported as mean ± SD in B. **p < 0.01, unpaired *t* test. **(C–F)** HeLa cells were transfected as in Figure 1A and analyzed by WB 4 days after transfection with indicated Abs. In **(C,E,F)**, representative WBs are shown. The asterisk marks aspecific band. In D, protein expression of indicated proteins was quantified, normalized to GAPDH and reported as fold relative to siCtr as mean ± SD of two experiments. In **(G)**, HeLa^Ctr–Cas9^ (Ctr-Cas9) and HeLa^HIPK2–Cas9^ (HIPK2-Cas9) were transfected with CEP55-specific (siCEP55) or control (siCtr) siRNAs, and MBR quantitation was performed by using ALIX as MBR markers in combination with β-tubulin costaining and DAPI to stain DNA and reported as in Figure 1D.

The autophagic receptors NBR1 and p62 and the CEP55/NBR1 complex have a major role in the autophagy-mediated degradation of MBRs (Pohl and Jentsch, 2009; Kuo et al., 2011; Isakson et al., 2013). In particular, CEP55 overexpression has been proposed to induce MBR accumulation by sequestering its interactor NBR1 in the cytoplasm (Kuo et al., 2011). To more directly evaluate whether HIPK2 regulates MBR fate by affecting the autophagy-mediated MBR degradation, we assessed both autophagy activity and the protein levels of p62, NBR1, and CEP55 in siCtr and siHIPK2 cells. Autophagy activity was evaluated by measuring the changes in the levels of LC3-II (the autophagic flux) in the presence or absence of CQ to inhibit lysosomal degradation and prevent the turnover of the LC3-II pool present in the autophagomes (Klionsky et al., 2008; Kuo et al., 2011). Compared with siCtr cells, the HIPK2-depleted cells showed an impairment of the autophagic flux (Figures 4A,B), supporting a role for HIPK2 in autophagy. In agreement with this result, we observed lower levels of p62 and NBR1 and higher levels of CEP55 in the HIPK2-depleted cells compared with the control cells (Figures 4C,D and Supplementary Figures S3A,B), whereas no major differences where observed for other midbody and MBR proteins, such as ALIX and TGS101 (Figures 4E–F). Finally, because of the key role of CEP55 in MBR degradation, we verified whether CEP55 depletion might impair MBR accumulation in HIPK2-depleted cells. To avoid multiple siRNA transfection, transient CEP55 depletion was induced in HIPK2-null cells (HeLa^HIPK2–Cas9^) generated by CRISPR/Cas9-mediated gene editing and in their related controls (HeLa^Ctr–Cas9^) (Ritter and Schmitz, 2019). In these HIPK2-null cells, the increase of CEP55 levels (Supplementary Figure S4A) and of MBR accumulation (Figure 4G, siCtr columns) was lower compared with the acutely depleted HeLa cells. Still, CEP55 depletion reduced the basal amount of MBRs in the Ctr-Cas9 cells and strongly inhibited MBR accumulation in the HIPK2-Cas9 (Figure 4G).

Altogether, these data suggest a model in which HIPK2 regulates MBR fate by acting on the autophagic pathway, both modulating the amount of the autophagic receptors p62 and NBR1 and modulating the activity of NBR1 through its ligand CEP55.

## Discussion

Unexpected new means of cellular communications depend on MBR fate, whose regulation is still largely unclear. Here, we show that the multifunctional kinase HIPK2, which localizes at the midbody and controls abscission, might regulate MBR fate by stimulating MBR removal via autophagic degradation.

It has been observed that differentiated cells mainly undergo symmetric bilateral abscission releasing MBR in the extracellular milieu, and it has been proposed that such event is critical to support a differentiation program by eliminating MBR intracellular signaling or by promoting the release of extracellular cues (Marzesco et al., 2005; Ettinger et al., 2011; Kuo et al., 2011). Stem-like populations and cancer cells, instead, appear more prone to undergo asymmetric abscission and to accumulate MBRs (Ettinger et al., 2011; Kuo et al., 2011). Our findings obtained both in tumor cells and in non-transformed RPE-1 cells show that HIPK2 depletion induces MBR accumulation, whereas depletion of the two known HIPK2 cytokinesis targets, Spastin and ecH2B, has only a marginal effect in MBR accumulation. From one side, these results indicate that the HIPK2 activities at midbody are not required for the following fate of the MBR. Alternatively, it can be speculated that both targets of HIPK2 are required to regulate the MBR fate. However, the observation that at least ecH2B depletion increases CEP55 levels at the midbody (LM and SS, unpublished results) favors the first hypothesis and supports the major role for CEP55 in both midbody and MBR regulation. In agreement, we observed that CEP55 depletion in HIPK2-null cells inhibits MBR accumulation. Whether this is due to a rescue of the autophagy activity or it is only linked to the CEP55-NBR1 interaction has to be defined.

HIPK2 has been shown to regulate the expression of a large amount of proteins by acting as cotranscriptional regulator of several promoters or by phosphorylation-dependent modulation of protein stability (Calzado et al., 2007; Rinaldo et al., 2007; Blaquiere and Verheyen, 2017). Based on the increased expression of CEP55 observed in both HIPK2- and ecH2B-depleted cells, we might speculate that HIPK2 controls CEP55 expression at the posttranscriptional levels. However, this hypothesis needs to be specifically addressed. Furthermore, at this point, we cannot exclude that HIPK2 contributes to MBR degradation through direct MBR subcellular localization.

Mechanistically, in different HIPK2-depleted/-null contexts, we found a change in the levels of CEP55 and of the main autophagic receptors involved in autophagy. This, along with the observed autophagic flux reduction, which is in the range of reduction reported between cancer and normal cells (Kuo et al., 2011), supports the hypothesis that HIPK2 modulates the MBR autophagy-mediated degradation. However, we cannot rule out an effect of HIPK2 depletion at least in part also on processes regulating MBR internalization or the choice between asymmetric or symmetric abscission, promoting asymmetric abscission and consequently MBR retention or symmetric abscission and MBR engulfment.

High levels of MBR have been correlated with enhanced tumorigenicity (Kuo et al., 2011; Dionne et al., 2017), and a causal relationship between them and cell proliferation, anchorage independent growth, and survival has been recently demonstrated (Peterman et al., 2019). Most evidence supports HIPK2 as a tumor suppressor, and others suggest HIPK2 as an oncogene and its role in tumor formation/progression appears complex and heterogeneous. In mouse, in transformed fibroblasts and in cancer cells of different origins, HIPK2 inactivation leads to increased tumorigenicity (D’Orazi et al., 2012; Feng et al., 2017). Even if it is difficult to separate effects caused by HIPK2-mediated regulation of MBR persistence from those resulting from the other HIPK2-dependent pathways, MBR regulation might be among the oncosuppressive functions attributed to this kinase. Further work will be needed to better understand and discriminate the consequences of HIPK2 dysfunctions leading to higher MBR levels on cancer formation/progression.

To deeply understand the controversial role of HIPK2, it will be interesting to explore whether HIPK2 is a broad regulator of autophagy. Autophagy consists of several stages, such as initiation, expansion, autophagosome formation, autophagosome–lysosome fusion, and degradation. Each of these steps can be dynamically regulated via a complex network of autophagy-relevant proteins and by posttranslational modifiers including kinases (Stork et al., 2012). It has been suggested that HIPK2 overexpression promotes phagophore expansion and increases mature autophagosome formation in liver sepsis (Jiang et al., 2018). In *Caenorhabditis elegans*, the HIPK-family orthologous HPK-1 is required for inducing autophagosome formation and autophagy gene expression in response to dietary restriction or TORC1 inactivation (Das et al., 2017). Here, we show that HIPK2 depletion impairs the autophagic flux and affects the levels of two major macroautophagy cargo receptors, p62 and NBR1, supporting the hypothesis that HIPK2 is one of the kinases involved in autophagy signaling, which impinges on MBR degradation also indirectly by acting on CEP55 expression.

Autophagy has been implicated not only in cancer but also inneurodegeneration, where autophagy signaling modulation is consideredan attractive field of therapeutic intervention. In particular, NBR1 is involved in ensuring ubiquitinated protein degradation, whose inappropriate aggregation is a common feature to numerous neurodegenerative and neuromuscular diseases (Nicot et al., 2014). HIPK2 has been involved in several neurodegenerative diseases, such as Parkinson, Alzheimer, and Lafora disease, mainly because of its role in proapoptotic induction (Lanni et al., 2010; Upadhyay et al., 2015). The HIPK2 role in autophagy regulation opens a scenario in which this kinase might function in the control of cell death and of protein aggregation. Of relevance, we observed a reduction of NBR1 levels and an increase of CEP55 levels also in HIPK2-depleted motoneuron-like cells and in brain tissue from HIPK2 KO mice (Supplementary Figures S3A,B and data not shown), supporting the existence of this HIPK2-mediated regulation in neuronal context both *in vitro* and *in vivo*. Thus, it will be interesting to explore mechanisms underlying this novel HIPK2 regulation and its role in normal and pathological conditions.

In conclusion, our data support a model in which, at the end of cell division, HIPK2 controls abscission acting on midbody regulation and MBR removal, this latter at least through autophagy-mediated degradation with clinical implication in cancer and in neurodegenerative diseases.

## Data Availability Statement

The datasets generated for this study are available on request to the corresponding authors.

## Ethics Statement

The animal study was reviewed and approved by National Institutes of Health (1056/2015).

## Author Contributions

CR designed the experiments and wrote the manuscript with the contribution of SS and FS. FS, LM, FM, and MF performed the experiments. CR and SS jointly supervised this work as co-last authors. All authors contributed to the article and approved the submitted version.

## Conflict of Interest

The authors declare that the research was conducted in the absence of any commercial or financial relationships that could be construed as a potential conflict of interest.

## Supplementary Material

FIGURE S1 MBR accumulation in HIPK2-depleted U2OS cells. **(A–C)** U2OS cells were transfected with HIPK2 -specific siRNA or negative universal control as in 1A and analyzed 5 days post transfection by WB with indicated Abs to verify RNAi and by IF for MBR quantitation as in 1C. Representative WB is shown in **A**. In **B**, the percentage of cells with ≥1 MBR were reported as mean ± SD by analyzing at least total 2000 cells per condition. ^∗∗^*p* < 0.01 and ^∗^*p* < 0.05, unpaired *t*-test. In **C**, representative fields are shown. Scale bar, 10 μM.

FIGURE S2 MBR upon depletion of HIPK2 and its cytokinesis target Spastin in non-transformed cells. **(A,B)** RPE-1 cells were transfected with HIPK2 -specific, Spastin-specific or negative control siRNAs and analyzed 5 days post transfection by WB with indicated Abs to verify RNAi and by IF for MBR quantitation. Representative WB are shown in **(A)**. In **(B)**, MBR quantitation is reported as mean ± SD by analyzing at least total 1000 cells per condition. Representative images are reported in the left panels. Scale bar, 10 μM. ^∗∗^*p* < 0.01, unpaired *t*-test.

FIGURE S3 NBR1 and CEP55 levels in HIPK2 depleted motorneuron-like cells and *in vivo* in *HIPK2* KO mouse. **(A)** NSC34 cells were transfected with a mix of three murine HIPK2 -specific siRNA or negative universal control and analyzed 4 days post transfection by WB with indicated Abs. **(B)** Cerebellum tissues were explanted from *HIPK2* KO or WT adult mice, protein lysates were obtained and analyzed by WB with indicated Abs. Protein expression of indicated proteins was quantified, normalized to GAPDH and reported below each WB.

FIGURE S4 CEP55 modulation in Ctr-Cas9 and HIPK2-Cas9 HeLa cells. **(A)** An equal number of Ctr-Cas9 and HIPK2-Cas9 HeLa cells were plated and TCEs were analyzed by WB with indicated Abs 24 h post plating. **(B)** Representative WB to verify CEP55 RNAi in cells analyzed in Figure 4G. At variance with Supplementary Figure S4A, the amount of CEP55 observed in the Ctr-Cas9 and HIPK2-Cas9 cells upon transfection with control siRNAs is similar. This is possibly due to the different confluence the cells reach after 4 days in culture (see panel in **C**), because of the abscission defects only in the HIPK2-Cas9 cells. **(C)** Representative images of the cells analyzed in Figure 4G. Scale bar, 10 μM.

TABLE S1 Cytokinesis defects were measured by IF in the indicated cells. DAPI was used to mark nuclei and β-tubulin immunostaining was used to identify midbody in telophase and cytoplasm in interphase, as described in Pisciottani et al., 2019 and Monteonofrio et al., 2019. Aberrant midbodies are those filled with microtubules, elongated, and often associated with tubulin-labeled puncta, as previously described in Pisciottani et al., 2019. Data are reported as mean ± SD from three independent experiments, in which at least 500 cells per condition were analyzed.
