# Supplementary figures and images for: HIPK2 Is Required for Midbody Remnant Removal Through Autophagy-Mediated Degradation
Source: Front Cell Dev Biol. 2020 Sep 15;8:572094. doi: 10.3389/fcell.2020.572094 (PMC7525647; doi:10.3389/fcell.2020.572094)

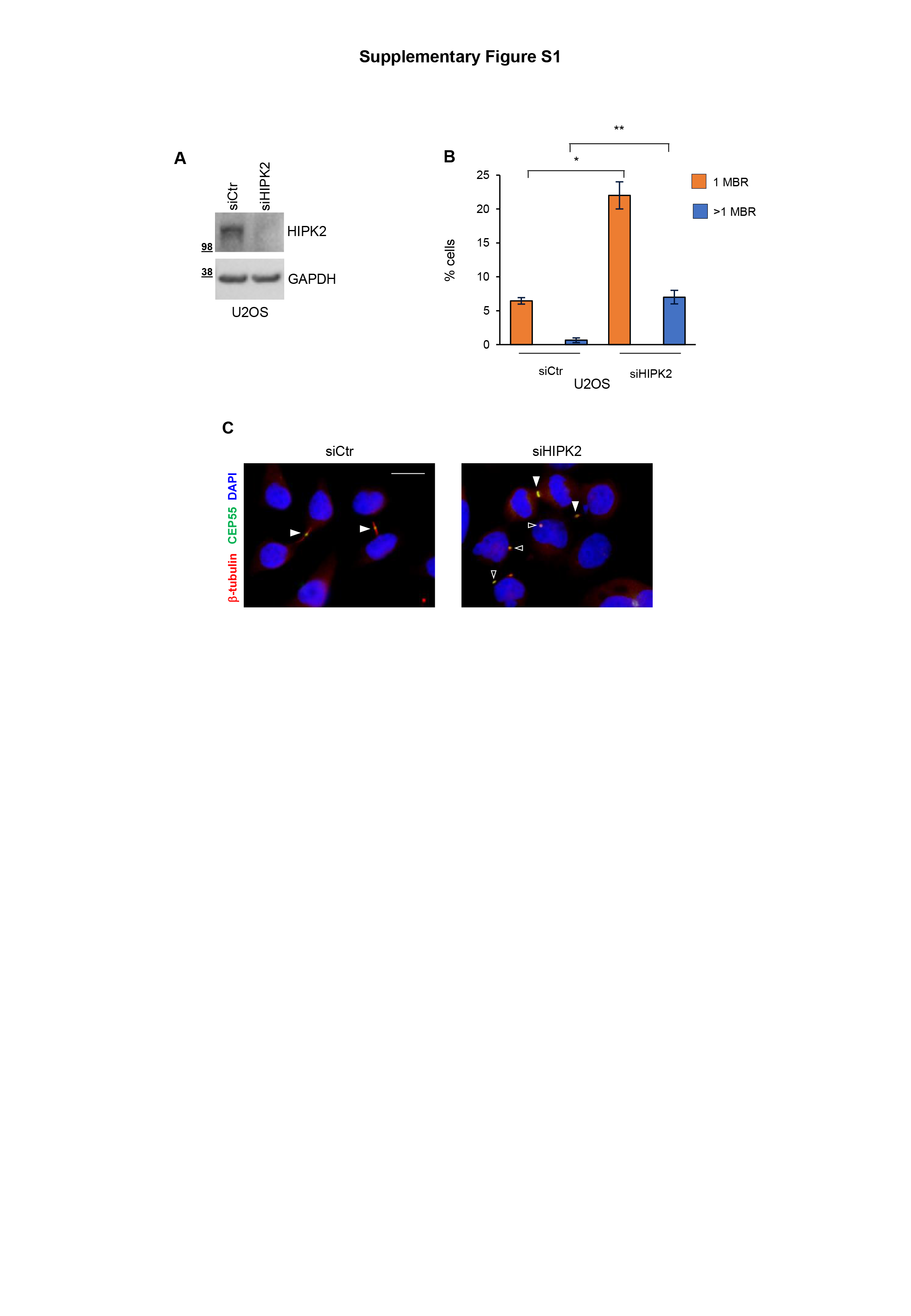

Supplement: FIGURE S1 — MBR accumulation in HIPK2-depleted U2OS cells. (A–C) U2OS cells were transfected with HIPK2 -specific siRNA or negative universal control as in 1A and analyzed 5 days post transfection by WB with indicated Abs to verify RNAi and by IF for MBR quantitation as in 1C. Representative WB is shown in A. In B, the percentage of cells with ≥1 MBR were reported as mean ± SD by analyzing at least total 2000 cells per condition. ∗∗p < 0.01 and ∗p < 0.05, unpaired t-test. In C, representative fields are shown. Scale bar, 10 μM. [file Image_1.TIF]

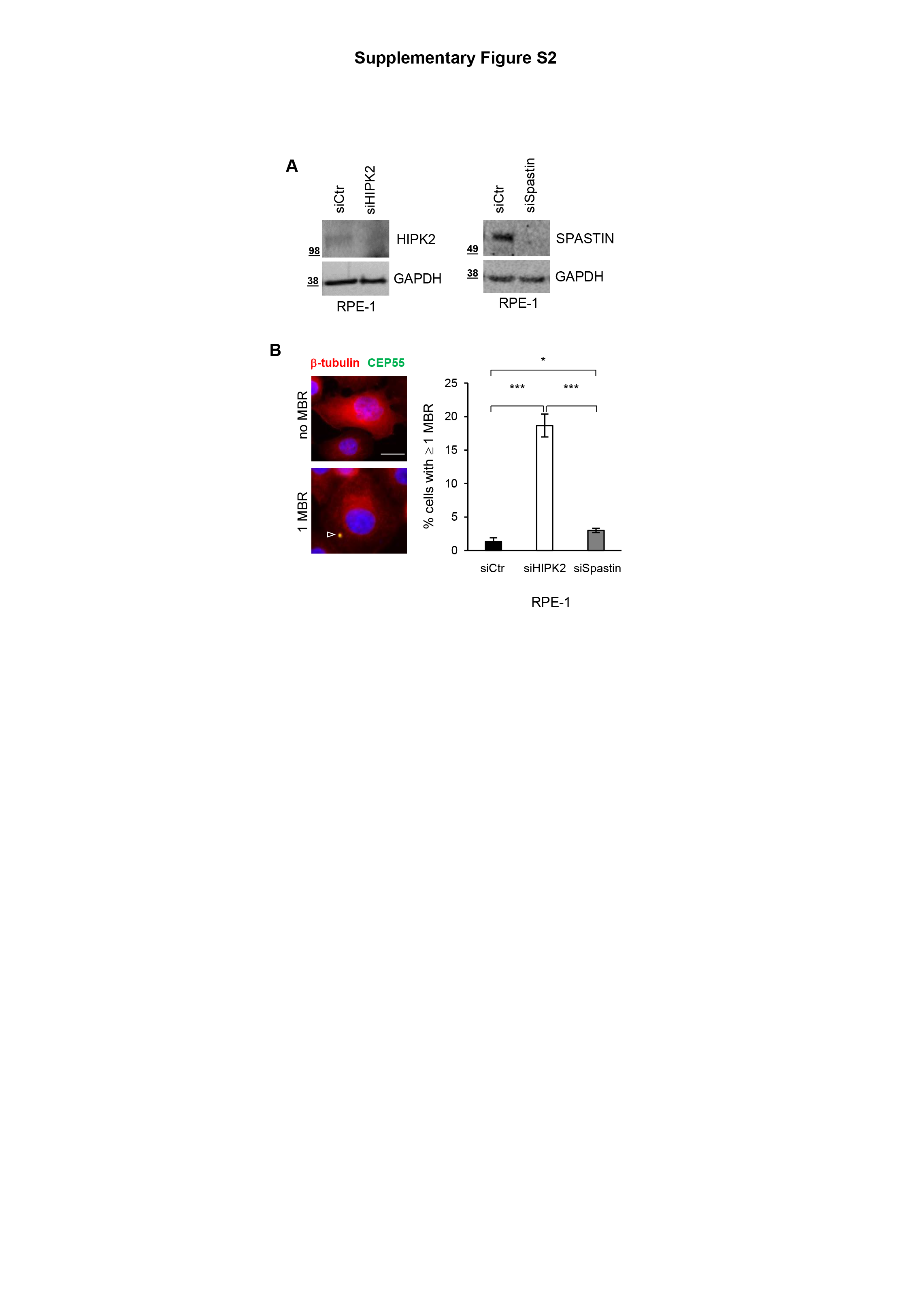

Supplement: FIGURE S2 — MBR upon depletion of HIPK2 and its cytokinesis target Spastin in non-transformed cells. (A,B) RPE-1 cells were transfected with HIPK2 -specific, Spastin-specific or negative control siRNAs and analyzed 5 days post transfection by WB with indicated Abs to verify RNAi and by IF for MBR quantitation. Representative WB are shown in (A). In (B), MBR quantitation is reported as mean ± SD by analyzing at least total 1000 cells per condition. Representative images are reported in the left panels. Scale bar, 10 μM. ∗∗p < 0.01, unpaired t-test. [file Image_2.TIF]

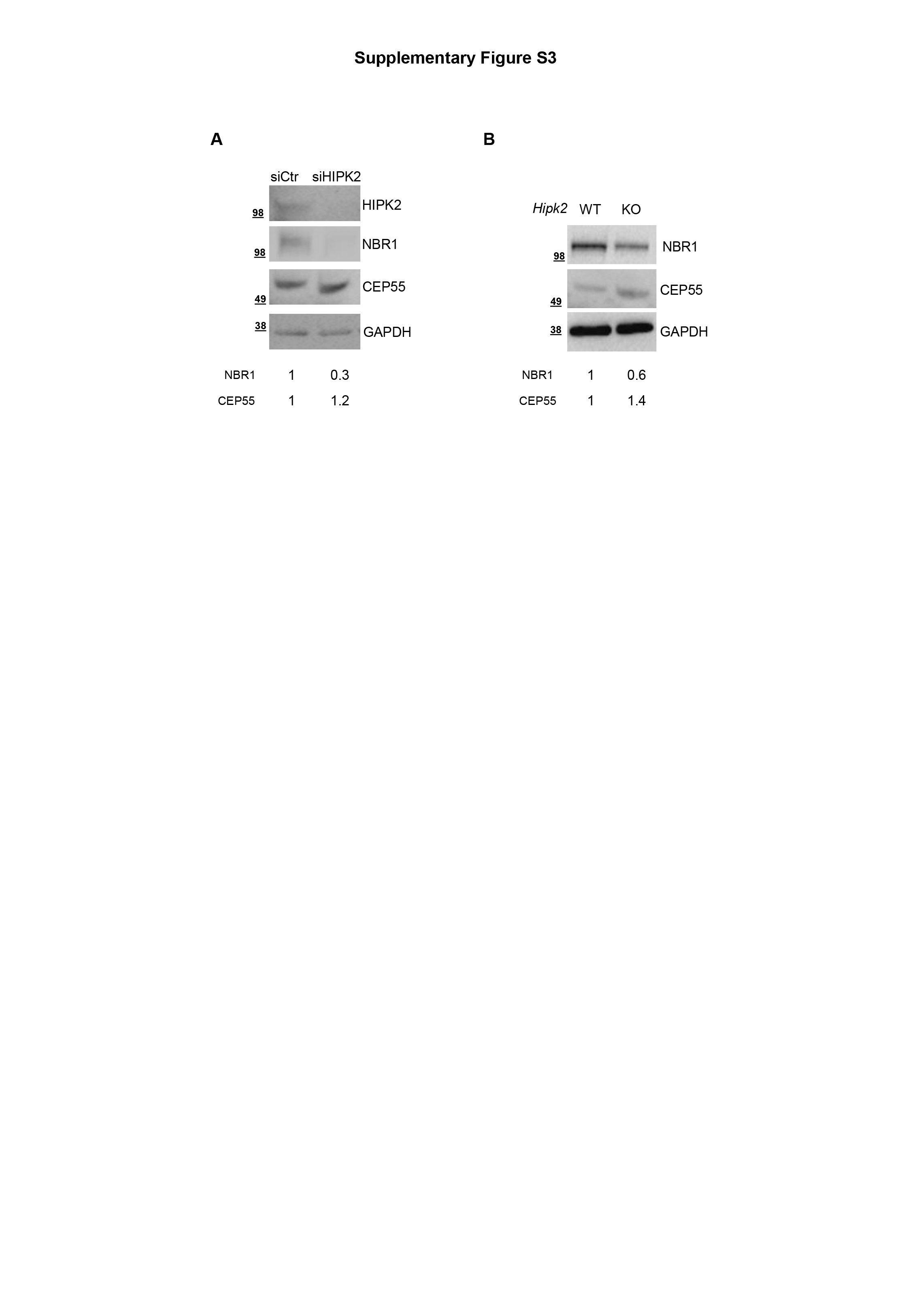

Supplement: FIGURE S3 — NBR1 and CEP55 levels in HIPK2 depleted motorneuron-like cells and in vivo in HIPK2 KO mouse. (A) NSC34 cells were transfected with a mix of three murine HIPK2 -specific siRNA or negative universal control and analyzed 4 days post transfection by WB with indicated Abs. (B) Cerebellum tissues were explanted from HIPK2 KO or WT adult mice, protein lysates were obtained and analyzed by WB with indicated Abs. Protein expression of indicated proteins was quantified, normalized to GAPDH and reported below each WB. [file Image_3.TIF]

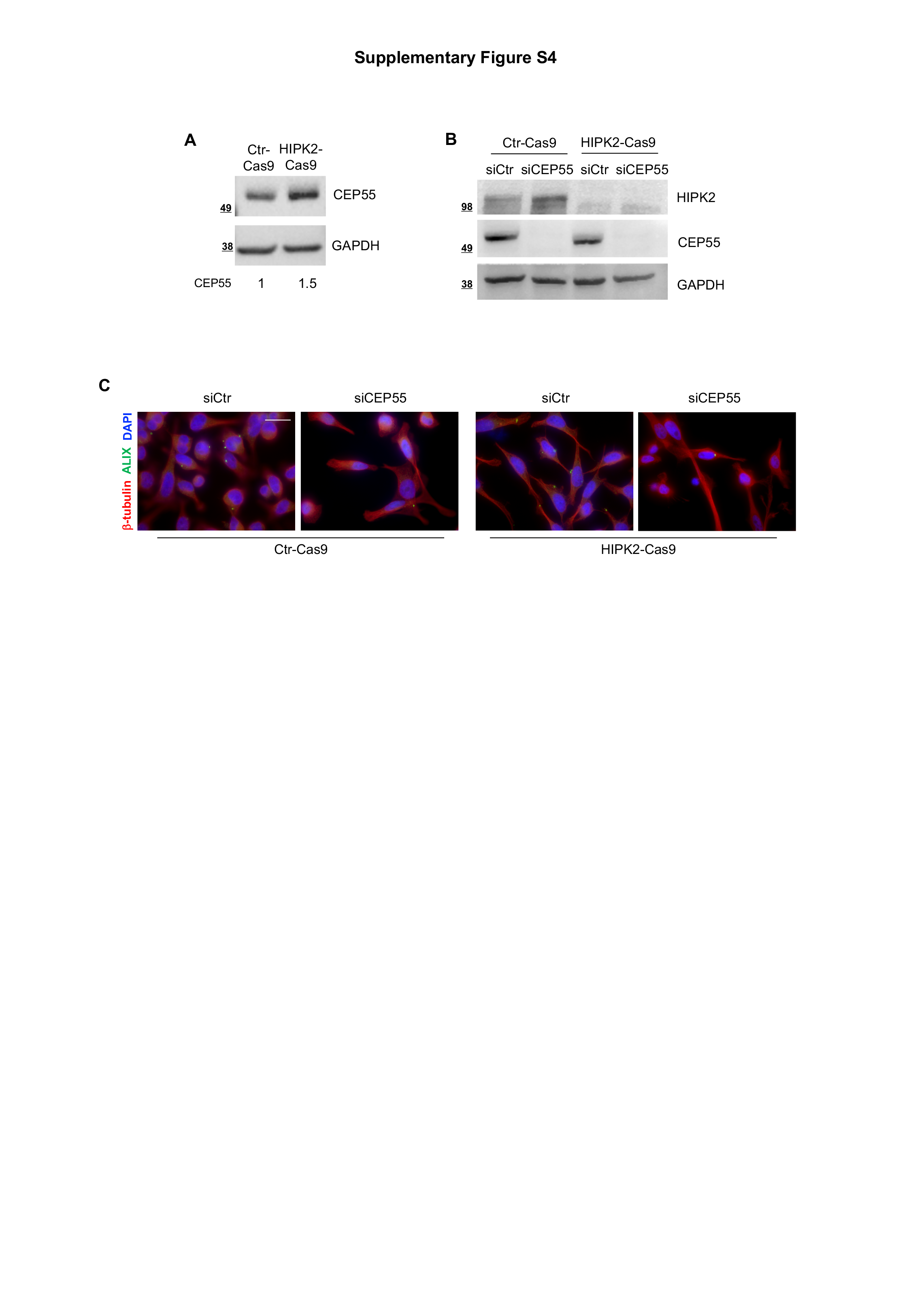

Supplement: FIGURE S4 — CEP55 modulation in Ctr-Cas9 and HIPK2-Cas9 HeLa cells. (A) An equal number of Ctr-Cas9 and HIPK2-Cas9 HeLa cells were plated and TCEs were analyzed by WB with indicated Abs 24 h post plating. (B) Representative WB to verify CEP55 RNAi in cells analyzed in Figure 4G. At variance with Supplementary Figure S4A, the amount of CEP55 observed in the Ctr-Cas9 and HIPK2-Cas9 cells upon transfection with control siRNAs is similar. This is possibly due to the different confluence the cells reach after 4 days in culture (see panel in C), because of the abscission defects only in the HIPK2-Cas9 cells. (C) Representative images of the cells analyzed in Figure 4G. Scale bar, 10 μM. [file Image_4.TIF]
