# Supplementary material for: HIPK2 Is Required for Midbody Remnant Removal Through Autophagy-Mediated Degradation
Source: Front Cell Dev Biol. 2020 Sep 15;8:572094. doi: 10.3389/fcell.2020.572094 (PMC7525647; doi:10.3389/fcell.2020.572094)
Supplement: TABLE S1 — Cytokinesis defects were measured by IF in the indicated cells. DAPI was used to mark nuclei and β-tubulin immunostaining was used to identify midbody in telophase and cytoplasm in interphase, as described in Pisciottani et al., 2019 and Monteonofrio et al., 2019. Aberrant midbodies are those filled with microtubules, elongated, and often associated with tubulin-labeled puncta, as previously described in Pisciottani et al., 2019. Data are reported as mean ± SD from three independent experiments, in which at least 500 cells per condition were analyzed. [file Table_1.pdf]

Supplementary Table S1

	CYTOKINESIS DEFECTS	
	**Aberrant midbodies (%)**	**Binucleated cells (%)**
siCtr	4.7 ± 2.1	2.1 ± 1.1
**siHIPK2**	**64 ± 6**	**17 ± 2.3**
siCtr	6.7 ± 1.2	3.3 ± 1.6
**siSpastin**	**75 ± 6.7**	**5 ± 1.2**
siCtr	9.1 ± 2.9	2.2 ± 1.1
**si-ecH2B**	**34.8 ± 3.7**	**2.7 ± 0.8**
